# Genetic ablation of Lmp2 increases the susceptibility for impaired cardiac function

**DOI:** 10.3389/fmolb.2024.1148948

**Published:** 2024-03-07

**Authors:** Felix A. Trogisch, Franziska Koser, Synje Michel, David A. Liem, Bogdan I. Florea, Markus Hecker, Oliver Drews

**Affiliations:** ^1^ European Center for Angioscience, Department of Cardiovascular Physiology, Mannheim Medical Faculty, Heidelberg University, Mannheim, Germany; ^2^ Department of Cardiovascular Physiology, Institute of Physiology and Pathophysiology, Heidelberg University, Heidelberg, Germany; ^3^ German Centre for Cardiovascular Research (DZHK), Partner Site Heidelberg/Mannheim, Heidelberg, Germany; ^4^ Departments of Physiology and Medicine/Cardiology, David Geffen School of Medicine, University of California, Los Angeles, Los Angeles, CA, United States; ^5^ Leiden Institute of Chemistry, Leiden University, Leiden, Netherlands; ^6^ Biomedical Mass Spectrometry, Center for Medical Research, Johannes Kepler University, Linz, Austria

**Keywords:** proteostasis, ubiquitin-proteasome system, cardiac remodeling, heart failure, proteasome intervention, adverse cardiac effects

## Abstract

Proteasome degradation is an integral part of cellular growth and function. Proteasomal intervention may mitigate adverse myocardial remodeling, but is associated with the onset of heart failure. Previously, we have demonstrated that increasing abundance of cardiac Lmp2 and its incorporation into proteasome complexes is an endogenous mechanism for proteasome regulation during hypertrophic remodeling of the heart induced by chronic *ß*-adrenoreceptor stimulation. Here, we investigated whether Lmp2 is required for myocardial remodeling not driven by inflammation and show that Lmp2 is a tipping element for growth and function in the heart but not for proteasome insufficiency. While it has no apparent impact under unchallenged conditions, myocardial remodeling without Lmp2 exacerbates hypertrophy and restricts cardiac function. Under chronic *ß*-adrenoreceptor stimulation, as seen in the development of cardiovascular disease and the manifestation of heart failure, genetic ablation of Lmp2 in mice caused augmented concentric hypertrophy of the left ventricle. While the heart rate was similarly elevated as in wildtype, myocardial contractility was not maintained without Lmp2, and apparently uncoupled of the *ß*-adrenergic response. Normalized to the exacerbated myocardial mass, contractility was reduced by 41% of the pretreatment level, but would appear preserved at absolute level. The lack of Lmp2 interfered with elevated 26S proteasome activities during early cardiac remodeling reported previously, but did not cause bulk proteasome insufficiency, suggesting the Lmp2 containing proteasome subpopulation is required for a selected group of proteins to be degraded. In the myocardial interstitium, augmented collagen deposition suggested matrix stiffening in the absence of Lmp2. Indeed, echocardiography of left ventricular peak relaxation velocity (circumferential strain rate) was reduced in this treatment group. Overall, targeting Lmp2 in a condition mimicking chronic *ß*-adrenoreceptor stimulation exhibited the onset of heart failure. Anticancer therapy inhibiting proteasome activity, including Lmp2, is associated with adverse cardiac events, in particular heart failure. Sparing Lmp2 may be an avenue to reduce adverse cardiac events when chronic sympathetic nervous system activation cannot be excluded.

## 1 Introduction

A network of perfectly tuned molecular mechanisms maintains protein homeostasis. The depth of its plasticity seems still at the beginning of our understanding. The ubiquitin proteasome system (UPS) precisely targets and degrades individual proteins as a mechanism of their regulation as well as aberrant proteins as a checkpoint of quality control (Labbadia and Morimoto, 2015; Collins and Goldberg, 2017; Pohl and Dikic, 2019). The number of proteins involved in the UPS exceeds more than 700 distinct members, which are encoded by a surprisingly large proportion of the total human genome (Hutchins et al., 2013). Proteasome complexes themselves are a heterogenic group with regulatory plasticity and harbor about 40 distinct subunits (Huang et al., 2016; Schweitzer et al., 2016; Collins and Goldberg, 2017). From a medical perspective, inhibiting proteasome activities provides promising therapeutic outcomes and is an established anti-cancer medication (Thibaudeau and Smith, 2019).

In heart disease, the dynamic regulation of the UPS can decide over the outcome of disease manifestation and progression (Drews and Taegtmeyer, 2014). In fact, the induction of heart failure is associated with proteasome inhibition in anti-cancer therapy (Cornell et al., 2019; Plummer et al., 2019). Furthermore, proteasome inhibition for a few months can be the primary cause of cardiac dysfunction in a controlled experimental setting (Herrmann et al., 2013). Cardiac proteasome activities are reduced in human heart failure and recover after ventricular unloading (Kassiotis et al., 2009; Predmore et al., 2010). Albeit these studies suggest avoiding proteasome inhibition in the context of cardiac disease, it has been demonstrated that during early cardiac remodeling with concentric hypertrophy, proteasome activities are increased (Depre et al., 2006; Drews et al., 2010). In this context, proteasome inhibition reverses cardiac remodeling and improves cardiac function (Hedhli et al., 2008; Stansfield et al., 2008). These and other reports suggest that proteasome inhibition requires a more precise understanding of proteasome activities and their regulation to avoid adverse cardiac events.

Proteasome inhibitors with approval for therapy in humans target the subunits, which confer the 20S proteasome with its proteolytic activities (Thibaudeau and Smith, 2019). In total, seven distinct subunits (β1, β2, β5, Lmp2/β1i, Mecl1/β2i, Lmp7/β5i and β5t) can be incorporated into 6 structural positions of a barrel-shaped 20S proteasome complex (28 subunits), which confer different preferences for amino acids flanking the cleavage site (Driscoll et al., 1993; Gaczynska et al., 1993; Murata et al., 2007). To date, the understanding of their individual role is limited. Lmp2, Mecl1 and Lmp7 are induced by proinflammatory signaling, such as interferon-*γ* (Gaczynska et al., 1993), and participate in antigen processing (Basler et al., 2013). These proinflammatory proteasome subunits participate and drive inflammatory cardiac disease (Bockstahler et al., 2020). In anti-cancer therapy, inhibiting the chymotrypsin-like proteasome activity is the primary target (Thibaudeau and Smith, 2019). Proteasome subunits contributing to chymotrypsin-like proteasome activity are β5, Lmp2 and Lmp7, which means they cleave preferentially after hydrophobic amino acid residues (Driscoll et al., 1993; Gaczynska et al., 1993). Altogether, Lmp2 is an inducible proteasome subunit, which drives inflammatory cardiac disease and is among the targeted catalytic subunits of anticancer therapy.

The incorporation of proteolytic subunits in 20S proteasome complexes and their abundance is dependent on cell-type and organ (Besse et al., 2019). Cardiac tissue contains lower active proteasome per total protein than other type of organs, potentially leaving the heart particular vulnerable to therapeutic intervention with proteasome inhibitors (Zong et al., 2006; Besse et al., 2019). The majority of 20S proteasome complexes in the heart incorporate the subunits β1, β2 and β5 and to a lower extend Lmp2, Mecl1 and Lmp7 (Zong et al., 2006; Drews et al., 2007), which canonically replace the former during proteasome complex assembly (Nandi et al., 1997). Interestingly, 20S proteasome complexes of mixed assembly exist in both heart and other cell types and organs, demonstrating a wide spectrum of plasticity and therapeutic targets (Drews et al., 2007; Klare et al., 2007; Drews and Taegtmeyer, 2014). Whether Lmp2 is required for cardiac function under conditions not primarily driven by inflammation is not known.

Dynamic proteasome assembly is a mechanism impacting proteasome function (Driscoll et al., 1993; Gaczynska et al., 1993). Previously, we demonstrated that Lmp2 contributes to proteasome regulation during cardiac remodeling not driven by inflammation (Drews et al., 2010). Here, we aimed at elucidating whether reduced 20S proteasome plasticity caused by lack of Lmp2 impacts cardiac remodeling and function under the influence of chronic activation of the *ß*-adrenergic system, which is a prevalent contributor to the development of heart failure (Hunter and Chien, 1999; Foody et al., 2002; Lohse et al., 2003). We hypothesized that proteasome subpopulations harboring Lmp2 decisively contribute to cardiac disease manifestation at cellular level, potentially impacting organ function.

## 2 Materials and methods

### 2.1 Animal experiments

All animal protocols were reviewed and approved (35-9185.81/G-194/11) by animal welfare authorities of the Heidelberg University as well as the State of Baden-Württemberg, Germany, and were conducted in agreement with the “Directive 2010/63/EU of the European Parliament and of the Council of 22 September 2010 on the protection of animals used for scientific purposes”. Animals were maintained with food and water *ad libitum* in a controlled environment. Male mice with a partial deletion of the *Psmb9* gene encoding Lmp2 and replacement/insertion of a neomycin resistance gene (*neo*) (Van Kaer et al., 1994) were used for this study (in the following referred to as Lmp2 knockout/KO mice; kind gift from L. Van Kaer, Vanderbilt University School of Medicine, Nashville, TN, United States). Upon rederivation, Lmp2 KO mice were backcrossed regularly with C57BL/6J wildtype (WT) mice. Homozygous WT and KO littermates according to *Psmb9* and *neo* genotyping via PCR were utilized for experiments (Supplementary Figure S1). At the age of approx. 12 weeks, the analysis was commenced by characterizing left ventricular morphology and function in the systole and diastole using echocardiography (parasternal long/PSLAX and short axis/SAX) as well as electrocardiography with a Vevo 2100 System equipped with a MS550D linear array transducer (VisualSonics) under mild isoflurane anesthesia (1%–2% in oxygenized ambient air; flow rate approx. 1 L/min). The following day, an osmotic minipump (Alzet, 1002) was implanted subcutaneously as described earlier under 2%–3% isoflurane anesthesia (Drews et al., 2010). Pumps were filled with either phosphate buffered saline (PBS) or 30 mg kg^−1^ d^−1^ isoproterenol in PBS (Sigma-Aldrich, I5627). Concentration of isoproterenol was based on batch-specific release rates of minipumps and animal weight. During anesthesia, body temperature was maintained at 37°C using a temperature-controlled pad while monitoring the temperature. Four and 7 days post implantation, electro- and echocardiography was repeated. An experimental summary is provided in Supplementary Figure S4. All parameters were analyzed in PSLAX M-mode recordings in Vevo Lab 5.6.2, except for circumferential peak strain rates, which were analyzed in SAX B-mode acquisitions with the integrated Vevo Strain Analysis suite. After 7 days, hearts were removed after euthanasia, perfused with PBS, weighted and quickly frozen in liquid nitrogen before storage at −80°C. For analyses by histology/immunofluorescence, hearts were additionally washed in 2,3-butanedione monoxime for 2 min and subsequently embedded in OCT (Tissue-Tek).

All analyses were performed in randomized parallel experiments and in a blinded manner. The latter means that experimenter and software user were unaware of the genotype as well the content of the minipumps (PBS or isoproterenol).

### 2.2 Cardiac sample preparation

Hearts were homogenized under non-denaturating conditions according to previously described protocols (Gomes et al., 2006; Drews et al., 2010). Briefly, hearts were manually homogenized in 1.5 mL buffer containing 20 mM HEPES (pH 7.5), 150 mM NaCl, 1 mM MgCl_2_, 0.5 mM EDTA, 1 mM DTT, and 1% phosphatase inhibitors (Sigma-Aldrich, P5726 and P0044) by 25 turns in a hand-held Tenbroeck tissue grinder (Wheaton) to obtain a crude lysate. Cytosolic fractions were obtained after ultracentrifugation at 100,000 × *g* for 60 min. All steps were performed on ice or at 4°C.

### 2.3 Proteasome activity assays

Cytosolic fractions were used to measure caspase-like, trypsin-like and chymotrypsin-like 26S proteasome activities as described previously (Gomes et al., 2006; Drews et al., 2010). Briefly, 25 µg cytosolic protein were assayed in a total volume of 100 µL homogenization buffer containing 50 μM ATP and using the following probes: Z-LLE-AMC (Calbiochem, 53914), Boc-LSTR-AMC (Bachem, I-1940), Suc-LLVY-AMC (Bachem, I-1395). The proteasome inhibitors Z-Pro-Nle-Asp-H (Enzo Life Sciences, BML-ZW9490) and epoxomicin (Boston Biochem, I-112) were utilized to determine non-proteasome activities. Fluorescence was measured on a FluoroSkan Ascent fluorometer (ThermoFisher) with an excitation wavelength of 390 nm and an emission wavelength of 460 nm every 5 min at 37°C for 90 min. Every microplate included an AMC standard curve. Specific proteasomal activites were derived from the linear phase of the fluorescence intensity curve and subtracting activities in presence of inhibitors from parallel measurements of total activity. All samples were measured in three technical replicates. Bar charts were normalized to groups assayed in parallel and shown as percentages for improved visualization.

### 2.4 Activity-based proteasome labeling

Cytosolic fractions were used for labeling of proteasome subunits with proteolytic activity with the activity-based probe MVB003 according to previously published protocols (Florea et al., 2010). Briefly, 25 µg cytosolic protein were used for activity-based labeling in homogenization buffer containing 50 μM ATP and 0.5 μM MVB003 at 37°C for 1 h. The proteasome inhibitor bortezomib was added at a concentration of 100 nM prior to activity based labeling for 1h at 4°C. Samples were separated by SDS-PAGE (12.5% acrylamide) and imaged on a Pharos FX scanner (Bio-Rad). Total protein was stained by ruthenium II tris (bathophenanthroline disulfonate).

### 2.5 Qualitative and quantitative analyses by Western blotting and immunofluorescence

SDS-PAGE, Western blotting (WB) and immunofluorescence (IF) analyses were conducted following standard protocols. The following antibodies and dyes were used: mouse anti-α7 (Enzo Life Sciences, PW8110, WB: 1:2500), mouse anti-α-actinin (Sigma-Aldrich, A7811, IF: 1:1500), mouse anti-β1 (Abcam, ab58081, WB: 1:1666), mouse anti-Lmp2 (Enzo Life Sciences, PW8840, WB: 1:800), rabbit anti-collagen I (Abcam, ab34710, IF: 1:150), rabbit anti-collagen III (Abcam, ab7778, IF: 1:300), rabbit anti-desmin (Dianova, DLN-13734, IF: 1:400), rabbit anti-lysine_48_-polyubiquitinated proteins (Merck Millipore, 05-1307, WB: 1:2500), donkey anti-mouse-Cy3 (Dianova, 715-165-151, IF: 1:1000), donkey anti-rabbit-Cy5 (Dianova, 711-175-152, IF: 1:500), goat anti-mouse-HRP (Abcam, ab98717, WB: 1:10,000), goat anti-rabbit-HRP (Abcam, ab97051, WB; 1:20,000); DAPI (Life Technologies, D1306, 1:5000), phalloidin-AF488 (ThermoFisher, A12379, 1:40), WGA-AF488 (ThermoFisher, W11261, 1:500). For WB of proteasome subunits, cytosolic extracts were supplemented with cOmplete Mini protease inhibitor cocktail (Roche) plus 50 µM bortezomib (LC Laboratories, B-1408). Lysine48-polyubiquitinated proteins were analyzed in crude lysates supplemented with protease inhibitor cocktail plus 50 µM bortezomib. Similar amounts of protein were loaded on 12.5% or 8% polyacrylamide gels and subsequently blotted onto nitrocellulose transfer membranes. Equal loading was validated via Ponceau S staining and utilized for normalization. ECL detection was performed by a LAS Mini 4000 imager (GE Healthcare). Quantification of WBs was performed via ImageQuant TL. Cryosections were prepared at 14 µm thickness and processed according to standard protocols. Same IF stainings were performed in parallel. An IX81 confocal microscope (Olympus) coupled to a CCD camera (Orca ER, Hamamatsu) was used to visualize IF staining. To determine CM cross section and length, either 100 cardiomyocytes or CMs in 6 fields of view at ×20 magnification were averaged per animal. Calibration was performed using a micrometer standard. For relative quantification of collagen I and III, imaging was performed in batches per staining at fixed magnification (×20), laser intensity and camera exposure. Measurements of at least 3 fields of view were averaged per animal. Microscopic images were analyzed using cellSens software. Bar charts were derived of averages per animal.

### 2.6 Statistical analyses

For statistical analysis, GraphPad Prism software (version 8, GraphPad Software Inc.) was used. Data is shown as means ± standard error of mean (s.e.m.). The number of biological replicates is indicated in the figure legends. An unpaired, two-tailed Student’s t-test was used to determine statistical significance when two groups were compared. A 2-way ANOVA and Fisher’s LSD *post hoc* test was utilized to account for 2 variables. More than two variables were accounted for with a paired 3-way ANOVA and Fisher’s LSD *post hoc* test. All tests were performed two-tailed, *p* < 0.05 was considered statistically significant.

## 3 Results

### 3.1 Lmp2 is not essential for cardiac development and function under unchallenged conditions

**FIGURE 1:**
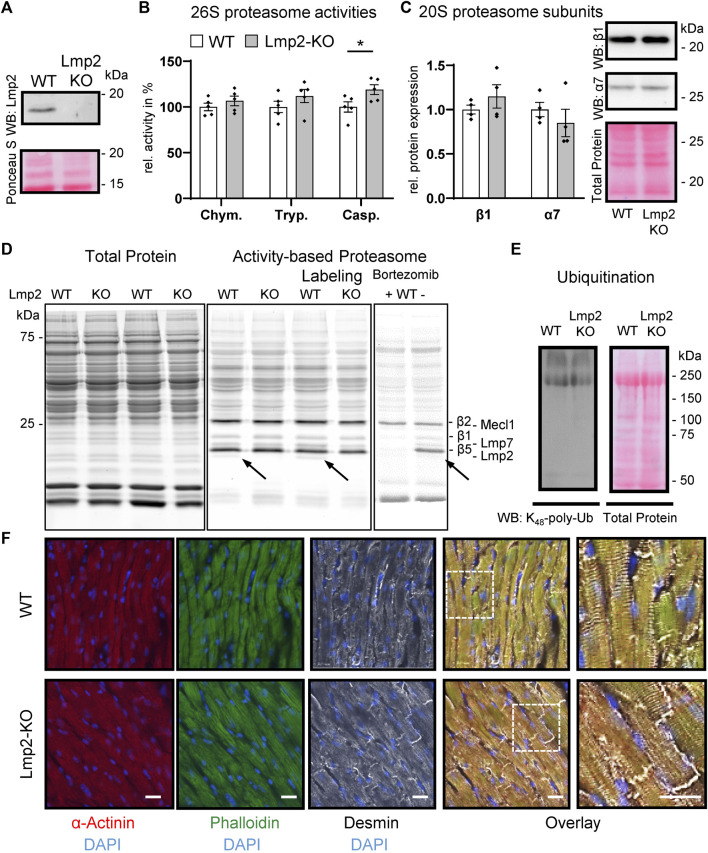
Cardiac expression of proteasome subunits and proteasome activities in presence and absence of Lmp2. Myocardial protein ubiquitination and sarcomere structure are not affected by Lmp2 expression. **(A)** Wildtype (WT) mice express predominantly the processed form of Lmp2 in the heart (approx. 20 kDa; precursor approx. 25 kDa), whereas no residual Lmp2 was detectable in knockout (KO) mice. Representative results of LMP2 immunodetection via WB. **(B)** Analysis of cardiac 26S proteasome activities. Chym.: chymotrypsin-, Tryp.: trypsin-, Casp.: caspase-like activity, *p < 0.05, mean ± s.e.m, *n* = 5, Student’s t-test. **(C)** Quantification and representative WB images of cardiac β1 and α7 abundance, *n* = 4. **(D)** Comparable levels of proteasome subunits conferring proteolytic activity. Proteasome assembly was assayed using activity-based proteasome labeling followed by SDS-PAGE and fluorescence detection of the activity-based probe. Representative results of 5 replicates are shown. Prior incubation with bortezomib interfered with proteasome labeling by blocking predominantly the subunits conferring chymotrypsin- and, to a lesser extent, caspase-like activity. Arrows indicate Lmp2. **(E)** WB of lysine_48_-poly-ubiquitinated proteins. Equal protein loading is displayed by total protein staining [Ruthenium in **(D)** and Ponceau S in **(A,C,E)**]. **(F)** Immunofluorescence of sarcomeric proteins and structure in cardiac cryosections. Scale bar: 20 µm. Representative results are shown.

For this study, Lmp2 constitutive knockout (KO) mice were backcrossed with wildtype (WT) C57BL/6J mice. Littermates of Lmp2 heterozygotes were validated to express Lmp2 by genotyping and in part by Western blotting (Figure 1A, Supplementary Figure S1). In Lmp2 KO mice, the corresponding proteasome subunit was not detectable. Development of adult Lmp2 KO mice was not associated with apparent differences in behavior, body weight and heart weight (Supplementary Table S1). Furthermore, morphometric and functional assessment *in vivo* by echocardiography showed no impact of Lmp2 KO on left ventricular posterior wall thickness (LVPW) and inner diameter (LVID) measured in diastole as well as left ventricular fractional shortening (LV-FS) and ejection fraction (LV-EF). Cardiac 26S proteasome activities in Lmp2 KO mice were comparable to WTs except for a 19% higher level of caspase-like activity (Figure 1B). The protein expression of other proteasome subunits and incorporation of catalytic proteasome subunits beside Lmp2 were not altered significantly (Figures 1C, D). The anticancer drug bortezomib inhibited predominantly cardiac proteasome subunits conferring chymotrypsin-like activity, including Lmp2 (Figure 1D). The minute difference in proteasome activity did neither affect the overall expression pattern of proteins in the heart nor did it affect the level and pattern of lysine_48_-poly-ubiquitinated proteins (Supplementary Figures S2, S3, Figure 1E). Furthermore, cardiac cryosections of Lmp2 KO mice did not show apparent differences in the staining pattern and intensity of *a*-actinin, phalloidin and desmin fluorescent immunostainings, indicating a normal development of sarcomere structures (Figure 1F). Altogether, the results demonstrate that the myocardium of Lmp2 KO mice is indistinguishable from that of WTs at macroscopic, functional and molecular level.

### 3.2 Absence of Lmp2 exacerbates cardiac hypertrophic remodeling towards a pathological phenotype

**FIGURE 2:**
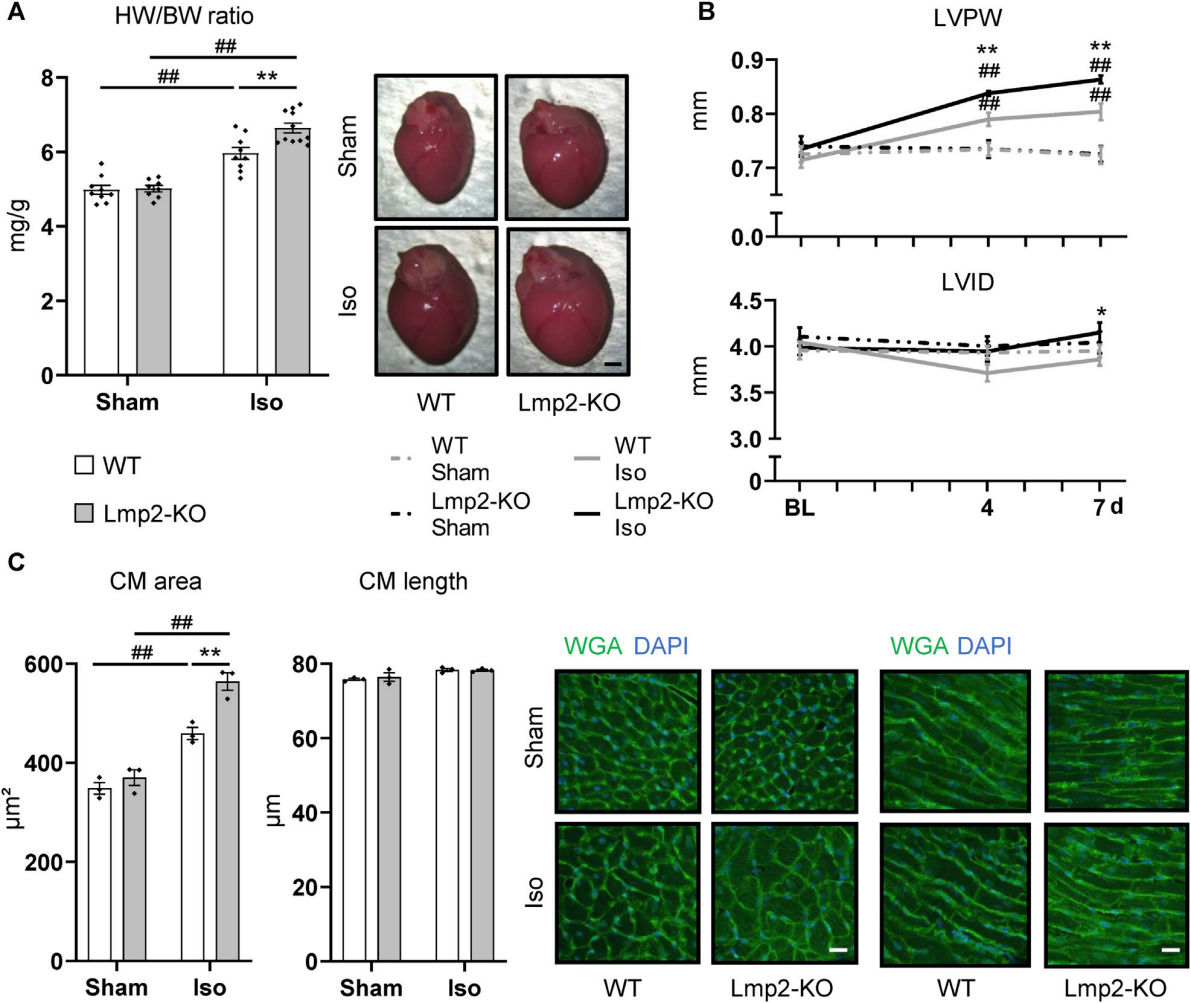
Isoproterenol induced exacerbated cardiac hypertrophy in absence of Lmp2. **(A)** Heart weight to body weight ratio (HW/BW) and representative images of whole hearts from wildtype (WT) and Lmp2 knockout (KO) mice after continuous sham or isoproterenol (Iso) treatment for 7 days. Scale bar: 1 mm **(B)** End-diastolic left-ventricular posterior wall thickness (LVPW) and internal diameter (LVID) measured by echocardiography. **(C)** Analysis of cross-sectional area and length of cardiomyocytes (CM) in wheat germ agglutinin (WGA) stained cryosections of hearts derived from wildtype (WT) and Lmp2 knockout (KO) mice after continuous sham or isoproterenol (Iso) treatment for 7 days. Representative images are shown. Scale bar: 20 µm, *p < 0.05, **p < 0.01 Iso WT vs. Iso Lmp2-KO, ^##^ *p* < 0.01 vs. corresponding sham, mean ± s.e.m., *n* ≥ 8 **(A, B)**, *n* = 3 mice **(C)**, 2-way ANOVA **(A, C)** or paired 3-way ANOVA **(B)** followed by Fisher’s LSD *post hoc* test.

Cardiac remodeling towards myocardial hypertrophy was investigated upon continuous *ß*-adrenoreceptor stimulation for up to 7 days via isoproterenol diffusion from a subcutaneously implanted osmotic minipump. Sham treated mice were subjected to the same procedure except the pumps contained PBS. Lmp2 WT mice responded to this treatment by a 20% increase in HW/BW ratio (Figure 2A). Unexpectedly, the HW/BW ratio increased by 32% in Lmp2 KO animals, which means an exacerbation of the response by 65% (parallel experiments to those with WT). Measuring cardiac remodeling *in vivo*, confirmed the observation of augmented cardiac hypertrophy in Lmp2 KO mice (Figure 2B). In fact, LVPW in Lmp2 KO mice was exacerbated already upon 4 days continuous *ß*-adrenoreceptor stimulation. Furthermore, the LVID in the diastole was increased moderately in Lmp2 KO mice. In contrast, LVID was rather decreased in WT animals.

At cellular level, cardiac remodeling was investigated via measuring cardiomyocyte cross-sectional area and length. Similar to the development of HW/BW ratio, cardiomyocyte area increased by 32% upon isoproterenol treatment in Lmp2 WTs, but was exacerbated 1.6-fold (52% vs. 32%) in Lmp2 KO mice (Figure 2C). In contrast, the length of cardiomyocytes was unaffected in both groups.

### 3.3 Absence of Lmp2 causes uncoupling of isoproterenol induced cardiac function and loss of contractility

**FIGURE 3:**
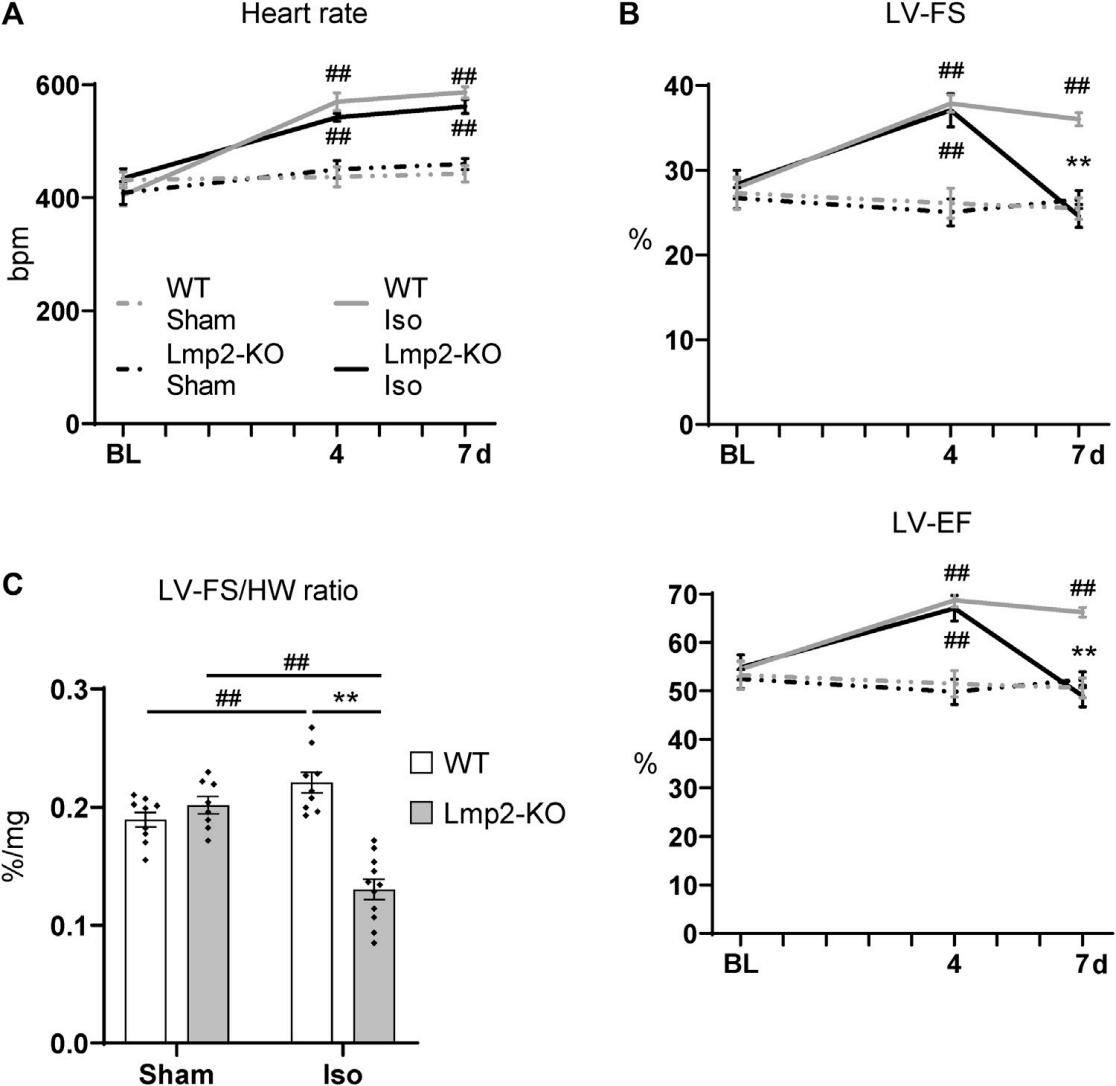
Lack of Lmp2 causes uncoupling of isoproterenol induced cardiac function, resulting in loss of contractility. Cardiac function was analyzed in wildtype (WT) and Lmp2 knockout (KO) mice after continuous sham or isoproterenol (Iso) treatment for 7 days. **(A)** Iso induced heart rate measured by ECG. **(B)** Iso induced left-ventricular fractional shortening (LV-FS) and ejection fraction (LV-EF) is followed by loss of both in Lmp2 KO mice; measured by echocardiography. **(C)** Hearts from Iso treated Lmp2 KO mice show less contractility per muscle mass compared to WT and sham treated animals. LV-FS to heart weight (HW) ratio as a measurement to represent contractility normalized to heart cardiac muscle mass. **p < 0.01 Iso WT vs. Iso Lmp2-KO, ^##^ *p* < 0.01 vs. corresponding sham, mean ± s.e.m., n ≥ 8, 2-way ANOVA (in C) or paired 3-way ANOVA (in A and B) followed by Fisher’s LSD *post hoc* test.

Cardiac function upon sham or continuous isoproterenol treatment was monitored by electro- and echocardiography (Supplementary Figure S4). Under baseline conditions, the heart rate of Lmp2 WT and KO mice showed no difference (Figure 3A, Supplementary Table S1). Both groups responded to isoproterenol by a similar increase in heart rate up to approx. 550 bpm, which was maintained until day 7. The heart rate of sham treated animals remained unaffected. Left ventricular systolic function paralleled the increase in heart rate in groups treated with isoproterenol for 4 days (WT +36%, Lmp2 KO +31% LF-FS), but diverged at day 7 (Figure 3B). WTs were able to maintain increased cardiac function, whereas Lmp2 KOs lost 34% of their LV-FS from day 4 to 7. Notably, LV-FS and -EF of Lmp2 KO mice decreased below baseline levels before the treatment (LV-FS 24.6% at 7d vs. 28.3% at BL; LV-EF 49% at 7d vs. 54.9% at BL). Altogether, the results show that isoproterenol induced heart rate and systolic function become uncoupled in absence of Lmp2 and contractility is lost unexpectedly.

Considering the severity in cardiac remodeling, the results show a substantial larger deficit of contractile function, because Lmp2 KO hearts harbor significantly more myocardial muscle mass (Figure 2) while performing poorly. This observation coincided at an experimental time point when they remained fully responsive to isoproterenol as indicated by the increased heart rate (Figure 3A). Normalizing LV-FS to the HW includes the aspect and shows that WTs have a LV-FS/HW ratio of 0.22%/mg whereas Lmp2 KOs perform at 0.13%/mg, which is a contractile deficit of 41% (Figure 3C).

### 3.4 Impaired hearts in challenged Lmp2 KO mice display reduced proteasome activities

**FIGURE 4:**
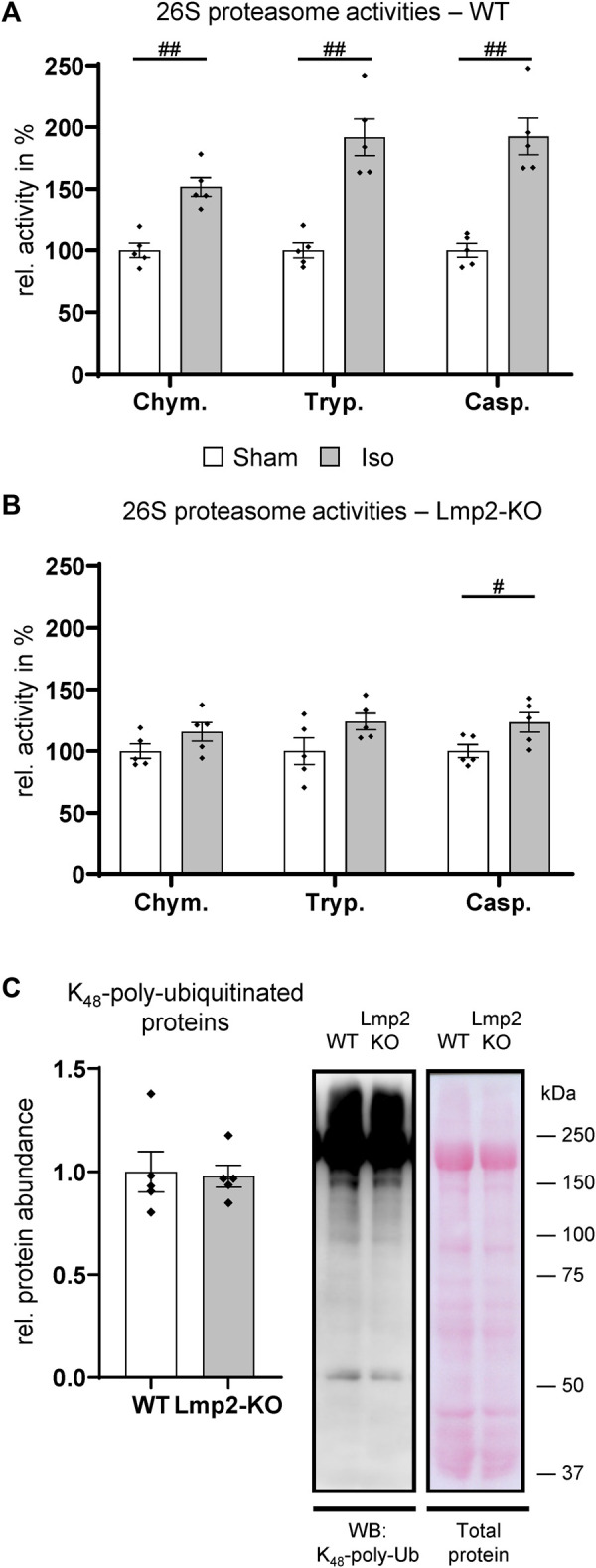
Hearts with reduced cardiac function fail to increase proteasome activities. The 26S proteasome activities were analyzed in wildtype (WT) and Lmp2 knockout (KO) mice after continuous sham or isoproterenol (Iso) treatment for 7 days. While hearts with increased LV-FS/HW in WT (Figure 3C) responded with a global increase in proteasome function compared to Sham **(A)**, those with reduced LV-FS/HW in Lmp2 KO showed only a moderate difference vs. Sham **(B)**. Chym.: chymotrypsin-, Tryp.: trypsin-, Casp.: caspase-like activity. **(C)** Global polyubiquitination of proteins was comparable in hearts of isoproterenol treated Lmp2 KO vs. WT mice. Immunodetection of lysine_48_-poly-ubiquitinated proteins in crude cardiac extracts of 7-day isoproterenol (Iso) treated animals by WB. Equal protein loading shown by total protein staining (Ponceau S). Representative images are displayed, #*p* < 0.05, ##*p* < 0.01 vs. corresponding sham mean ± s.e.m., *n* = 5, Student’s t-test.

In unchallenged mice, the lack of Lmp2 did not seem to have an apparent effect on cardiac contractility and a seemingly negligible effect on proteasome function (Figure 1B, Supplementary Table S1). In isoproterenol challenged hearts of WT mice, 26S proteasome activities were increased by 52%–92% depending on the type of proteolytic activity (Figure 4A). It should be noted that Figure 4 does not display the absolute, but relative proteasome activities, which were normalized to those in PBS treated animals. In the myocardium of isoproterenol treated mice, the chymotrypsin-like activity was with 176 ± 7.8 pmol substrate/(mg extract × min) the predominant activity. Trypsin- and caspase-like activities were 10 ± 0.7 and 25 ± 1.7 pmol/(mg×min), respectively.

In contrast to the substantial increase in treated WT mice, 26S proteasome activities were elevated only slightly in poorly performing hearts with a contractile deficit upon 7-day isoproterenol treatment due to the lack of Lmp2 (Figure 4B, Supplementary Table S2). The ratio of the three 26S proteasome activities in relation to each other remained similar as is WTs. In the myocardium of isoproterenol treated Lmp2 KO mice, the chymotrypsin-like activity remained with 136 ± 7.9 pmol substrate/(mg extract × min) the predominant activity. Accordingly, trypsin- and caspase-like activities were lower at 6 ± 0.3 and 21 ± 1.2 pmol/(mg × min), respectively. Analysis of lysine_48_-poly-ubiquitinated proteins in both groups showed that the failed enhancement of proteasome function did not cause those proteins to accumulate in general, suggesting no bulk proteasome insufficiency as a consequence of lacking Lmp2 (Figure 4C).

### 3.5 Deteriorated cardiac remodeling is associated with augmented interstitial matrix development

Poly-ubiquitinated proteins may accumulate in the cell as aggregates, which are poorly soluble. Therefore, we investigated the sarcomeric structure by immunofluorescence microscopy and included desmin, which is associated with deteriorating cardiac and proteasome function when it accumulates aberrantly in the myocardium. Intracellular protein aggregation in general and specifically of desmin was not associated with exacerbated hypertrophic remodeling and loss of contractile function in absence of Lmp2 (data not shown). Myofibrillar disarray was not observed.

**FIGURE 5:**
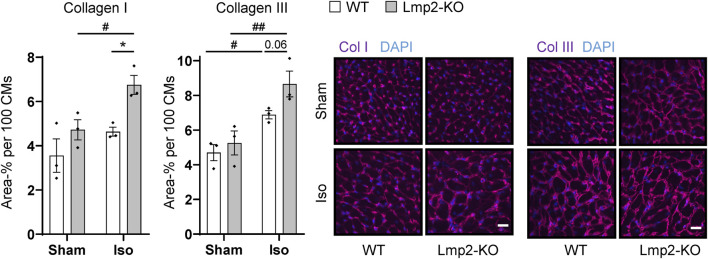
Isoproterenol induced interstitial deposition of collagen in hearts lacking Lmp2. Interstitial collagen (col) I and III deposition was analyzed by immunofluorescence in wildtype (WT) and Lmp2 knockout (KO) mice after continuous sham or isoproterenol (Iso) treatment for 7 days. Scale bar: 20 μm, *p < 0.05 Iso WT vs. Iso Lmp2-KO, ^#^ *p* < 0.05, ^##^ *p* < 0.01 vs. corresponding sham, mean ± s.e.m., *n* = 3 mice, number indicates non-significant *p*-value of interest, 2-way ANOVA and Fisher’s LSD *post hoc* test.

In contrast, a significant increase of the extracellular matrix proteins collagen I and III was observed in the interstitium of hearts with augmented hypertrophy induced by 7-day isoproterenol treatment in Lmp2 KO mice (Figure 5). Since the cardiomyocytes of those hearts have a larger cross-sectional area than in the WT background, the fluorescence intensity was normalized to 100 cardiomyocytes. Isoproterenol induced an overall increase of extracellular collagen I and III deposition in the myocardium of both Lmp2 WT (+30% Col I; +47% Col III) and KO mice (+43% Col I, +65% Col III). In Lmp2 KO, this increase was 41% larger for collagen I and 39% for collagen III though.

### 3.6 Isoproterenol induced augmented interstitial matrix development is accompanied by reduced peak strain rates

Increased collagen deposition in the extracellular matrix may be associated with its stiffening, which in turn can impact the velocity of myocyte motion to a level that it restricts cardiac function. LV-FS and -EF do not reflect this aspect properly as these experimental parameters are calculated without a temporal component. Strain rates measured via speckle tracking of echocardiography 2-D scans over time enable to calculate peak velocity.

**FIGURE 6:**
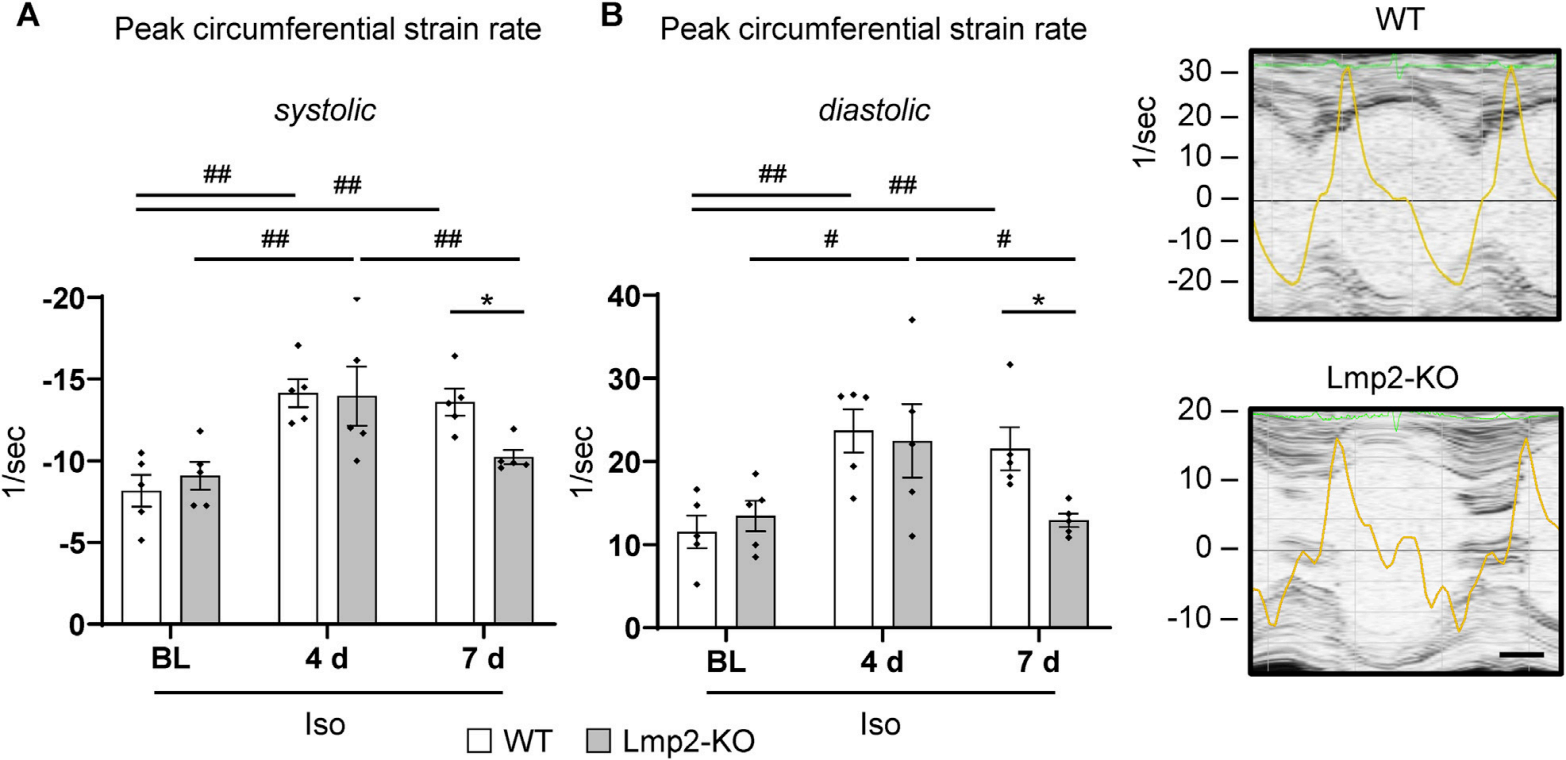
Lack of Lmp2 causes loss of isoproterenol induced peak strain rate in both myocardial contraction and relaxation. Peak circumferential strain rates during left ventricular contraction **(A)** and relaxation **(B)** were analyzed by echocardiography in wildtype (WT) and Lmp2 knockout (KO) mice after continuous isoproterenol (Iso) treatment for up to 7 days. While hearts in WT mice responded with a persistent increase in peak strain rate during both phases of the cardiac cycle, those in Lmp2 KO mice lost the gain of day 4 at day 7 of the treatment. Scale bar: 25 ms, representative short axis M-line echocardiograms including peak circumferential strain rate (yellow trace) for 7d Iso are shown, *p < 0.05 Iso WT vs. Iso Lmp2-KO, ^#^ *p* < 0.05, ^##^ *p* < 0.01 vs. time points as indicated, mean ± s.e.m., *n* = 5, paired 2-way ANOVA followed by Fisher’s LSD *post hoc* test.

At day 4 of continuous *ß*-adrenoreceptor stimulation, the myocardial peak circumferential strain rates in both Lmp2 WT and KO mice reached approx. −14/sec (Figure 6A). Upon day 7, WT mice maintained peak circumferential strain rates at elevated levels. In contrast, the Lmp2 KO group fell back to pretreatment levels. Force development is linked to myocardial contractility. Changes in contractility, as observed via loss of LV-FS (Figure 3B), largely contribute to altered peak circumferential strain rates during systole. Within this context it is interesting that peak circumferential strain rates during diastolic relaxation were similarly increased at day 4 of the treatment, but then reduced in Lmp2 KO animals at day 7 (Figure 6B). The reduced deformation rate during diastole occurred during the passive filling of the left ventricle (Figure 6B echocardiogram of Lmp2 KO). In those ventricles, aggravated interstitial deposition of collagen was observed (Figure 5), suggesting augmented accumulation of extracellular matrix contributed to the loss in peak diastolic circumferential strain rate.

## 4 Discussion

The composition of proteasome complexes and their activities are regulated dynamically to adapt to cellular requirements. Previously, we have demonstrated that a heterogeneous group of 20S proteasome subpopulations with distinct proteolytic activities exists in the myocardium (Drews et al., 2007). Furthermore, subpopulation composition and activities are regulated by increased incorporation of inducible proteasome subunits in the development of cardiac hypertrophy (Drews et al., 2010). In the current study, we provide first evidence that the inducible subunit Lmp2 is required to prevent an aggravated manifestation of concentric cardiac hypertrophy with reduced cardiac function and increased matrix development in a noninflammatory model. Proteasome function is impaired in heart failure and targeting proteasome activities is associated with a risk to develop cardiac adverse events, in particular heart failure (Drews and Taegtmeyer, 2014; Cornell et al., 2019; Plummer et al., 2019). Our results suggest a novel mechanism reducing the risk to develop heart failure by endogenous proteasome regulation via Lmp2 as a consequence of chronic activation of the *ß*-adrenergic system.

Lmp2 is a proteasome subunit with a special feature, because it changes cleavage site preference from caspase- (acidic residues) to chymotrypsin-like (hydrophobic) activity, when replacing its counterpart β1 during proteasome assembly (Driscoll et al., 1993; Gaczynska et al., 1993). Other subunits change the rate of substrate cleavage but not the preference for amino acids flanking the cleavage site (Coux et al., 1996; Thibaudeau and Smith, 2019). Thus, the slight increase in caspase-like proteasome activity in hearts of unchallenged Lmp2 KO mice can be explained by increased incorporation of β1. Since Lmp2 is incorporated in a small proportion of cardiac proteasome complexes (Zong et al., 2006; Drews et al., 2007), the effect on β1 incorporation into proteasome complexes and global proteasome activity is expected to be small as observed. It has been proposed that inducible subunits like Lmp2 facilitate antigen presentation, but more recently new roles with broader implications emerge (Angeles et al., 2012; Kruger and Kloetzel, 2012). Since hearts of Lmp2 KO mice exhibited normal cardiac morphology, microanatomy and function, our study solidifies the previous notion that Lmp2 is not required for embryonic and postnatal development as well as organ function under unchallenged conditions, albeit it impacts proteasome activities (Van Kaer et al., 1994; Cai et al., 2008).

Chronic *ß*-adrenoreceptor stimulation is an established contributor to the development of heart failure and the fundamental basis for pharmaceutical intervention with *ß*-blockers (Hunter and Chien, 1999; Foody et al., 2002; Lohse et al., 2003). The development of cardiac hypertrophy in response to pathophysiological stimuli appears compensatory, but has distinct features when compared to physiological hypertrophy (Hunter and Chien, 1999; Frey et al., 2004; Heineke and Molkentin, 2006). Increased thickening of cardiomyocytes without matching longitudinal growth is a feature of concentric hypertrophy. It is a stage in cardiac disease manifestation. Exacerbation of cardiac hypertrophy as seen in our results caused by the lack of Lmp2 would be considered a progression of disease development, which may lead to heart failure.

Short-term *ß*-adrenoreceptor stimulation enhances cardiac performance, such as heart rate and contractility, and was unaffected by Lmp2 in the present study. In contrast, long-term stimulation causes desensitization and loss of cardiac contractility (Rockman et al., 2002; Lohse et al., 2003). Lack of Lmp2 appears to accelerate the pathology of chronic *ß*-adrenoreceptor stimulation as shown by the loss of systolic function. Notably, this occurred while the heart rate remained elevated, meaning *ß*-adrenoreceptor signaling of heart rate was uncoupled from contractility. Since *ß*-adrenoreceptor stimulation increases cardiac Lmp2 abundance and its incorporation in proteasome complexes (Drews et al., 2010), it is potentially one additional therapeutic advantage of preserving *ß*-adrenoreceptor signaling, e.g., by *ß*-blockers. Whether the beneficial role of cardiac Lmp2 on cardiac remodeling can be augmented is an intriguing question.

Normalization of systolic function to the excessive cardiac mass in treated Lmp2 KO mice demonstrates a substantial loss of contractility beyond pretreatment levels albeit LV-EF would appear borderline preserved to mildly reduced from a clinical perspective (HFpEF: LV-EF ≥ 50%; HFmrEF LV-EF: 41%–49%) (Heidenreich et al., 2022). A recent clinical study suggests that the risk for adverse cardiac events, particularly heart failure, is rather common upon pharmacotherapy with proteasome inhibitors (Cornell et al., 2019). Notably, the majority of those patients experiencing heart failure with proteasome inhibitor-based therapy had preserved ejection fraction.

Under normal/unchallenged conditions, proteasome capacity is considered to be present in excess to maintain stable protein homeostasis (Dantuma and Lindsten, 2010). During early hypertrophic remodeling, this capacity is further enhanced by increasing 26S proteasome activities (Depre et al., 2006; Drews et al., 2010). Here, we see a depression of that effect in absence of Lmp2 and link it to deteriorated remodeling with reduced cardiac function. Human biopsies of dysfunctional hearts exhibit reduced proteasome activities (Kassiotis et al., 2009; Predmore et al., 2010; Day et al., 2013). Whether reduced proteasome activities are a contributing cause of heart failure or a consequence of a deteriorating cardiac function in humans is not fully understood. Several cardiac proteins can be degraded via the UPS (Drews and Taegtmeyer, 2014) and severe proteasome insufficiency may cause their accumulation and intracellular aggregation, which in turn may contribute to disease progression (Wang et al., 2011). Such an effect was not observed in the present investigation. Gross proteasome activities in failing hearts of Lmp2 KO mice were sufficient to prevent abnormal accumulation of lysine_48_-poly-ubiquitinated proteins. Furthermore, sarcomeric structure appeared similar to those in WT. Altogether, the results suggest that the reduced elevation of proteasome activities is rather a consequence of deteriorating cardiac function than the contributing cause. Instead, a Lmp2 containing proteasome subpopulation drives the pathology without apparent proteasome insufficiency.

Previous investigations suggest proteasome complexes containing inducible catalytic subunits degrade oxidatively damaged proteins without prior ubiquitination (Angeles et al., 2012; Kruger and Kloetzel, 2012; Raynes et al., 2016). In a prior study, we showed that oxidatively damaged proteins increase along with Lmp2 in the heart upon acute *ß*-adrenoreceptor stimulation, but return to basal levels beyond 24h treatment while cardiac proteasome activities were unchanged (Drews et al., 2010). Thus, we concluded that oxidative stress may contribute to the induction of Lmp2, but the myocardium adapted to the oxidative stress through a proteasome-independent pathway and proteasome regulation had no observable effect on the total level of oxidized proteins. A recent study suggests that adaptation of adult rat ventricular myocytes during *in vitro* cultivation requires Lmp2 to breakdown pre-existing sarcomeres (Petersen et al., 2020). Degradation by the UPS is a major pathway to breakdown sarcomere proteins (Martin and Kirk, 2020). More specifically, myosin heavy chain is reported to be predominantly degraded by proteasomes in neonatal rat ventricular myocytes upon interferon-γ stimulation (Cosper et al., 2012). In the current study, impaired or reduced degradation of an abundant sarcomere protein in absence of Lmp2 was not observed in form of altered total protein expression, accumulation of ubiquitinated proteins, protein aggregation or myofibrillar disarray. As a consequence, we conclude that the Lmp2 containing proteasome subpopulation is required rather for the degradation of a selected group of proteins instead of abundant cardiac components.

Fibrosis is an integral component of the pathology of heart disease (Khan and Sheppard, 2006; Travers et al., 2016), including those with persistent *ß*-adrenergic pathway stimulation (Kudej et al., 1997; Lohse et al., 2003). Here, we show augmented interstitial collagen deposition during exacerbated hypertrophic remodeling caused by the lack of Lmp2. Fibrosis and augmented interstitial collagen deposition cause stiffening of the heart, which impacts diastolic function and is associated with poor outcomes in heart failure with preserved ejection fraction (Khan and Sheppard, 2006; Ohtani et al., 2012; Travers et al., 2016). In the present investigation, diastolic function is enhanced during early *ß*-adrenoreceptor stimulation. In absence of Lmp2, the enhancement of diastolic peak strain rate is lost despite *ß*-adrenoreceptor stimulation is maintained as evidenced by increased heart rate. In those hearts, highest levels of interstitial collagen deposition were observed, which may restrict diastolic function.

Chymotrypsin-like proteasome activities are inhibited by systemic exposure with chemotherapeutics in anti-cancer therapy (Thibaudeau and Smith, 2019), which primarily affects subunits β5, Lmp2 and Lmp7 in the heart (Besse et al., 2019). Previous investigations link heart failure to proteasome function, but do not disclose whether proteasome inhibition in general or of a specific proteasome subunit contribute to cardiac disease development. Targeting β5 in the context of cardiac remodeling is associated with reduced hypertrophy but accelerated progression of heart failure and increased mortality (Ranek et al., 2015). Beyond the role of Lmp2 and Lmp7 in the immune response and antigen processing (Van Kaer et al., 1994; Kincaid et al., 2011; Kruger and Kloetzel, 2012) and their impact on inflammatory cardiac disease (Opitz et al., 2011; Bockstahler et al., 2020), their contribution to cardiac remodeling not driven by inflammation is unknown. In the present study, genetic ablation of Lmp2 exacerbated concentric hypertrophy, increased interstitial matrix deposition and reduced both systolic as well as diastolic function. Overall, we conclude that targeting cardiac Lmp2 should be avoided, because it deteriorates the capacity of the myocardium to cope with sustained *ß*-adrenergic signaling, resulting in abnormal cardiac remodeling and premature loss of cardiac function.

## Data Availability

The raw data supporting the conclusion of this article will be made available by the authors, without undue reservation.
