# Supplementary material for: Genetic ablation of Lmp2 increases the susceptibility for impaired cardiac function
Source: Front Mol Biosci. 2024 Mar 7;11:1148948. doi: 10.3389/fmolb.2024.1148948 (PMC10955435; doi:10.3389/fmolb.2024.1148948)
Supplement: Supplementary file 1 [file DataSheet1.pdf]

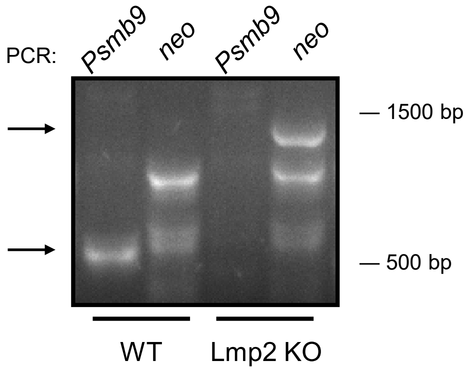

Supp. Fig. 1: Genotyping of littermates derived from heterozygote Lmp2 mice showing one specific band for *Psmb9* (bottom arrow; wildtypes) or *neo* (top arrow; knockout/KO).

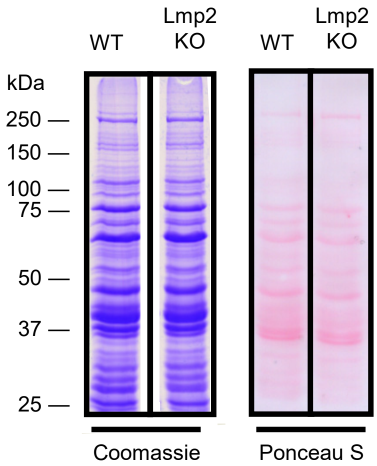

Supp. Fig. 2: Myocardial protein expression is not affected by Lmp2 expression. Overall cardiac expression of cytosolic proteins derived from wildtype (WT) and Lmp2 knockout (KO) mice.

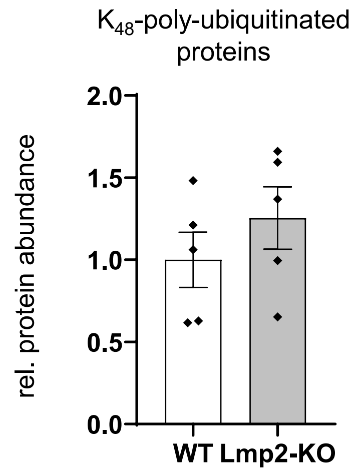

Supp. Fig. 3: Myocardial protein ubiquitination in wildtype (WT) and Lmp2 knockout (KO) mice is similar, mean±s.e.m., n=5. Representative WB shown in Fig. 1E.

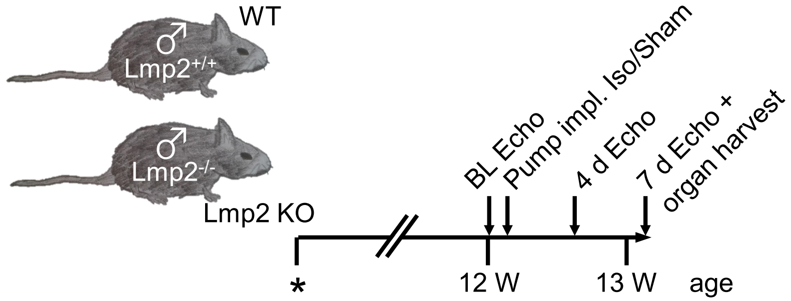

Supp. Fig. 4: Overview of the experimental design for the continuous isoproterenol (Iso) challenge of mice with wildtype (WT) and knockout (KO) Lmp2 background.

Supp. Tab. 1: Lmp2 is not essential for heart weight and function in adult mice under unchallenged conditions. HR, heart rate; LVPW, left-ventricular posterior wall thickness and LVID, left-ventricular internal diameter at end-diastole; %LV-FS, left-ventricular fractional shortening; %LV-EF, left-ventricular ejection fraction; all data are expressed as mean±s.e.m.; n≥10; † echocardiography of combined groups shown in Fig. 2B and Fig. 3A & B under baseline conditions (BL; before surgery and treatment) n≥18; see Supp. Fig. 4 for a timeline of analyses and treatments.

	**WT**	**Lmp2 KO**
Body weight (g)	25.8±0.8	25.8±1.1
Heart weight (mg)	131±6	134±6
HW/BW (mg/g)	5.1±0.1	5.2±0.1
HR (bpm)	418±13 †	424±14 †
LVPW (mm)	0.72±0.01 †	0.74±0.01 †
LVID (mm)	4.00±0.05 †	4.04±0.06 †
%LV-FS	27.6±1.0 †	27.6±1.1 †
%LV-EF	53.9±1.6 †	53.9±1.7 †

Supp. Tab. 2: Hearts with reduced cardiac function fail to increase proteasome activities. Three 26S proteasome activities were analyzed in wildtype (WT) and Lmp2 knockout (KO) mice after continuous sham or isoproterenol (Iso) treatment for 7 days. While hearts with increased LV-FS/HW in WT (Fig. 3C) responded with a global increase in proteasome function compared to Sham, those with reduced LV-FS/HW in Lmp2 KO showed only a moderate difference vs. Sham. The columns Δ and p_i show the difference in gained proteasome activity between the groups and the significance, mean±s.e.m., n=5, 2-way ANOVA.

**26S activity**	**WT Iso v Sham**	**Lmp2 KO Iso v Sham**	**Δ (Lmp2 KO - WT)**	**P_i**
Chymotrypsin-like	+ 51.7±7.5%	+ 15.7±7.5%	- 36.0	0.017
Trypsin-like	+ 91.7±14.8%	+ 24.0±6.7%	- 67.7	0.004
Caspase-like	+ 92.5±14.8%	+ 23.3±8.0%	- 69.2	0.002
